# Isolation and crystal and mol­ecular structures of [(C_5_H_2_Br_3_)_2_Fe], [(C_5_HBr_4_)_2_Fe] and [(C_5_Br_5_)(C_5_Br_4_HgBr)Fe]

**DOI:** 10.1107/S205322962200955X

**Published:** 2022-10-13

**Authors:** Tobias Blockhaus, Karlheinz Sünkel

**Affiliations:** a Chemistry, Ludwig-Maximilians-University Munich, Butenandtstrasse 5-13, Munich, D-81377, Germany; University of Sheffield, United Kingdom

**Keywords:** noncovalent inter­actions, halogen bonding, Hirshfeld analysis, crystal structure, bromo­ferrocene

## Abstract

The reaction of [(C_5_H_3_Br_2_)_2_Fe] with lithium tetra­methyl­piperidinide (LiTMP) in a 1:10 molar ratio in tetra­hydro­furan yields, after quenching with C_2_H_2_Br_4_, a mixture of the polybromo­ferrocenes [C_10_H_10–*n*_Br_*n*_Fe] with *n* = 4–9, from which single crystals of bis­(1,2,3-tri­bromo­cyclo­penta­dien­yl)iron(II), [Fe(C_5_H_2_Br_3_)_2_], and bis­(1,2,3,4-tetra­bromo­cyclo­penta­dien­yl)iron(II), [Fe(C_5_HBr_4_)_2_Fe], were obtained by a combination of chromatography and fractional crystallization. Treatment of ‘[C_10_(HgOAc)_10_Fe]’ with KBr_3_ yields a mixture of polybromo­ferrocenes [C_10_H_10–*n*_Br_*n*_Fe] with *n* = 8–10 and bromo­mercurioferrocenes [C_10_H_9–*n*_Br_*n*_(HgBr)Fe] with *n* = 7–9, from which single crystals of (1-bromo­mercurio-2,3,4,5-tetra­bromo­cyclo­penta­dien­yl)(1,2,3,4,5-penta­bromo­cyclo­penta­dien­yl)iron(II), [FeHgBr(C_5_Br_4_)(C_5_Br_5_)], were obtained by fractional crystallization. The crystal structures of all the com­pounds show Br⋯Br, Br⋯H and sometimes Br⋯Cp⋯π (Cp is a ring centroid) interactions, as well as π–π inter­actions. The findings are supported by Hirshfeld analyses.

## Abstract 2

Hexa- and octa­bromo­ferrocene, as well as a mercury derivative of deca­bromo­ferrocene, were isolated from mixtures of polybromo­ferrocenes and characterized by X-ray diffraction. The crystals show halogen and hydrogen bonding, which act co-operatively.

## Introduction

‘Noncovalent inter­actions’ are found in nearly all disciplines of chemistry, biochemistry and biology, and have been studied, at least in part, for quite a while (Hobza & Řezáč, 2016 ▸). This term brings together such apparently different inter­actions as hydrogen, halogen, lone-pair–π, anion–π, cation–π and π–π bonding, and these inter­actions can either act independently or co-operatively (Mahadevi & Sastry, 2016 ▸; Portela & Fernández, 2021 ▸). Among these, halogen bond(ing) has been studied continuously at a high level since about 1995. The last com­prehensive review dates back to 2016 (Cavallo *et al.*, 2016 ▸). A look at *SciFinder* shows since then nearly 2000 new entries for the years 2021 and 2022, and already 492 entries with the concept ‘Halogen Bonding’ (accessed on May 26th, 2022). The vast majority of these studies are centred on organic or biological systems, with a focus on crystal engineering (Mukherjee *et al.*, 2014 ▸). Relatively rarely studied were metal-containing systems (Brammer *et al.*, 2008 ▸), in particular, organometallic systems have so far been restricted to a few metal carbonyls, ruthenium-com­plexed aryl iodides (Kelly & Holman, 2022 ▸) and one study on 1,1′-dihaloferrocenes (Shimizu & Ferreira da Silva, 2018 ▸). Our group has been working on polyhalogenated metallocenes for quite a while (Sünkel & Motz, 1988 ▸; Sünkel & Hofmann, 1992 ▸; Sünkel *et al.*, 1994 ▸, 2015 ▸; Sünkel & Bernhartzeder, 2011 ▸), and some very recent reports on the synthesis and crystal structure determinations of [(C_5_H_*n*_Br_5–*n*_)(C_5_H_2_Br_3_)Fe] (*n* = 1 or 2; Butler *et al.*, 2021 ▸; Butler, 2021 ▸) and [(C_5_H_*n*_Br_5–*n*_)(C_5_Br_5_)Fe] (*n* = 0 or 1; Rupf *et al.*, 2022 ▸) prompted us to report on our synthetic and crystallographic studies of polybromo­ferrocenes. A special focus is made on the occurrence of halogen and hydrogen bonding in these systems.

## Experimental

### Synthesis and crystallization

#### Reaction of 1,1′,2,2′-tetra­bromo­ferrocene (1) with LiTMP in a 1:10 molar ratio and C_2_H_2_Br_4_

A solution of **1** (243 mg, 0.48 mmol) in tetra­hydro­furan (THF; 2 ml) was added to a freshly prepared solution of LiTMP (4.8 mmol) in THF (4 ml) at −30 °C. After stirring for 5 h, the temperature was lowered to −78 °C and C_2_H_2_Br_4_ (0.6 ml, 5.0 mmol) was added. With continuous stirring, the temperature was raised to ambient temperature over a period of 16 h. After this, water (10 ml) was added and the mixture was extracted with several 10 ml portions of CH_2_Cl_2_. The combined extracts were washed with water, then dried with MgSO_4_ and com­pletely evaporated *in vacuo*. The residue was taken up in the minimum amount of petroleum ether and chromatographed on an alumina column (20 × 2 cm), using petroleum ether as eluent. 21 fractions were collected and examined by mass spectroscopy and selected fractions were examined by ^1^H NMR spectroscopy (Figs. S1 and S2 in the supporting information). All fractions contained mixtures of poly­bromo­ferrocenes. While the first fraction consisted of a mix­ture of penta-, hexa- and hepta­bromo­ferrocene, the inter­­mediate fractions contained hexa-, hepta- and octa­bromo­ferrocene, and the last fraction was a mixture of hepta- and octa­bromo­ferrocene with traces of nona­bromo­ferrocene. Crystals of 1,1′,2,2′,3,3′-hexa­bromo­ferrocene (**3**) were ob­tained by slow evaporation of the sixth fraction in a refrigerator, and crystals of 1,1′,2,2′,3,3′,4,4′-octa­bromo­ferrocene (**5**) were obtained from the last fraction by the same method. All other fractions were also recrystallized from different solvents (petroleum ether, Et_2_O and CH_2_Cl_2_), but yielded neither crystals nor ‘pure’ powders (according to ^1^H NMR spectra taken after redissolution).

Hexa­bromo­ferrocene (**3**). ^1^H NMR (270 MHz, CDCl_3_): δ 4.47 ppm (literature: 4.47 ppm; Butler, 2021 ▸). MS (DEI): *m*/*z* = 659.6 (calculated 659.5).

Octa­bromo­ferrocene (**5**). ^1^H NMR (400 MHz, DMSO-*d*_6_): δ 5.20 ppm. MS (DEI): *m*/*z* = 817.4 (calculated 817.3).

#### Reaction of ‘[C_10_(HgOAc)_10_Fe]’ with KBr_3_

A suspension of ‘permercurated ferrocene’ (2.78 g, *ca* 1 mmol) with KBr_3_, freshly prepared from KBr (1.19 g, 10 mmol) and Br_2_ (0.512 ml, 10 mmol) in water (100 ml), was stirred for 4 h at room temperature. After filtration, the residue was first washed with water and then extracted with di­chloro­methane. The combined extracts were evaporated *in vacuo* and the residue was placed on top of an alumina column. A 1:1 mixture of petroleum ether and di­chloro­methane eluted two yellow bands. The first fraction consisted, according to its mass spectrum (Fig. S3), of a mixture of deca-, nona- and octa­bromo­ferrocene, while the second fraction yielded a mixture of the bromo­mercurioferrocenes [C_10_H_*n*_Br_9–*n*_HgBrFe] with *n* = 0–2 (Fig. S4). The ^1^H NMR spectrum of the first fraction showed four weak signals, which unfortunately could not be assigned to individual com­pounds (Fig. S5). Recrystallization attempts with the first fraction yielded again only mixtures, while from the second fraction, crystals of [(C_5_Br_5_)(C_5_Br_4_HgBr)Fe] (**8**) could be obtained.

### Refinement

Compound **3**: *SHELXT* (Sheldrick, 2015*a* ▸) provided the com­plete mol­ecule of **3** on the first run. The following difference Fourier synthesis (see Fig. S6 of the supporting information) showed two electron-density maxima (Q15 and Q16 in Fig. S6; *d* = 2.66 and 2.48 e Å^−3^) at radial distances of 1.54 and 1.40 Å from ring atoms C24 and C14, respectively. Despite these short distances (more typical for C—C bonds), we assigned these peaks to Br atoms (first named X1 and X2) with very low site-occupancy factors, since from the preceeding synthesis no other elements could have been present. The following refinement, however, showed rather short inter­molecular distances (2.581/3.328 and 2.955/3.106 Å) from these positions to atoms Br21^i^/Br22^i^ and Br11^i^/Br12^i^, respectively [symmetry code: (i) *x*, *y* − 1, *z*] (Fig. S7). It was con­cluded that X1/X2 could not be present in the same mol­ecule as Br11/Br12/Br21 (and eventually Br22 also) and therefore it was assumed that com­pound **3** (with Br11–Br13 and Br21–Br23) cocrystallized with very small amounts (*ca* 3%) of com­pound **2** [with Br13–Br14 (= X2) and Br22–Br24 (= X1)], and this model was used for the subsequent refinements. The refinement procedure was as follows: first, it was assumed that all Br atoms would have the same isotropic *U* values and then the site-occupation factors for X1 = Br24/H24 and X2 = Br14/H14, as well as Br11/H11, Br12/H12, Br21/H21 and Br23/H23, were allowed to refine. The site-occupancy factors were then fixed at these values and the *U* values were allowed to refine freely for the main com­ponents even anisotropically. Any attempts to produce longer C14—Br14 and C24—Br24 ‘bonds’ *via* the use of restraints met with failure. It should be noted at this point that the crystal structure of 1,1′,2,4-tetra­iodo­ferrocene showed a similar disorder and an apparent ‘bond shortening’, which the authors were able to resolve (Evans *et al.*, 2021 ▸).

Compound **5**: the measured crystal was recognized as a twin (two domains, rotated by 180° around 010) and a HKLF5 data file was created. The scale factor BASF refined to a final value of 0.17818. The refinement proceeded without any problems, and no signs of disorder were found.

Crystal data, data collection and structure refinement details of all com­pounds are summarized in Table 1 ▸.

## Results and discussion

### Synthesis

According to a recent review on haloferrocenes, there were only three heteroannularly substituted polybromo­ferrocenes known in 2018 (Butenschön, 2018 ▸): 1,1′-di­bromo­ferrocene, 1,1′,2-tri­bromo­ferrocene and deca­bromo­ferrocene. Since then, at least one isomer of each of the remaining [C_10_H_*n*_Br_10–*n*_Fe] with *n* = 4–9 has been obtained, sometimes only as part of mixtures. There were two different synthetic approaches to achieve this: (i) stepwise li­thia­tion followed by elec­tro­philic quenching with ‘Br^+^’, starting with 1,1′-di­­­bromo­ferrocene, or (ii) ‘permercuration’ of ferrocene followed by treatment with KBr_3_. Both methods had their shortcomings, however. When 1,1′-di­bromo­ferrocene was treated with 2.1 equivalents of LiTMP in THF at low tem­per­ature, followed by electrophilic quenching with 1,1,2,2-tetra­bromo­ethane, a mixture of tri-, tetra-, penta-, hexa-, hepta- and octa­bromo­ferrocenes was obtained, from which the first two could be obtained in pure form (yields of 9.9 and 16.0%, respectively; Butler *et al.*, 2021 ▸). When the solvent was changed from THF to hexane, the electrophile to di­bromo­hexa­fluoro­propane and the temperature to room temperature, 1,1′,2,2′-tetra­bromo­ferrocene (**1**) was obtained in over 90% yield (Butler, 2021 ▸). Repeating the latter procedure on com­pound **1** gave rather high yields of 1,1′,2,2′,3,3′-hexa­bromo­ferrocene (**3**), contaminated, however, with hepta­bromo­ferrocene (**4**) and octa­bromo­ferrocene (**5**). All attempts to repeat this procedure on com­pound **3** met with failure, due to the very low solubility of this com­pound. On the other hand, the preparation of ‘permercurated ferrocenes’ followed by the addition of KBr_3_, first reported in 1977, then later in 1994, 1997 and 2022, suffered from difficulties due to solubility problems (Boev & Dombrovskii, 1977 ▸; Han *et al.*, 1994 ▸; Neto *et al.*, 1997 ▸; Rupf *et al.*, 2022 ▸). For example, Han and co-workers showed that using Hg(O_2_CCF_3_)_2_ as the mercuration agent gave a ‘mixture of at least four partially brominated ferrocenes’. When they used Hg(O_2_CCH_3_)_2_ as the mercuration agent, deca­bromo­ferrocene (**7**) could be isolated in 60% yield, contaminated, however, with at least two partially brominated ferrocenes. Rupf and co-workers repeated this latter experiment and showed that besides **7** also nona­bromo­ferrocene (**6**) and nona­bromo(bromo­mer­cur­io)ferro­cene (**8**) were formed (based on ^13^C NMR spectroscopy; a closer look at Fig. S20 of their sup­porting information shows the additional formation of octa­bromo­ferrocene **5** and the bromo­mercurioferrocenes [C_10_H_*n*_Br_9–*n*_HgBrFe] with *n* = 1 and 2). When they used Hg(O_2_CC_3_H_7_)_2_, they apparently obtained a mixture of **7** and **8** with no other contaminants (based on NMR and IR). Neto and co-workers reported the use of Hg(O_2_CCCl_3_)_2_ as the mercuration agent, the transformation of the apparently formed [C_10_(HgO_2_CCl_3_)_10_Fe] to the deca­chloro­mercurio­ferrocene, followed by reaction with KBr_3_ to give pure **7** [characterization by NMR and IR spectroscopy, and elemental analysis (C and Fe)].

We decided to look at the li­thia­tion reactions with LiTMP as the li­thia­ting reagent, C_2_H_2_Br_4_ as the brominating agent and THF as the reaction medium at low temperatures again. We started with a solution of 1,1′,2,2′-tetra­bromo­ferrocene (**1**; purity > 95%) in THF and treated it with ten molar equivalents of LiTMP, followed by the addition of tetra­bromo­ethane. After standard work-up, a chromatographic separation was attempted. Since no band formation was recognizable, 21 fractions with equal volume were collected. Fractions 1, 10, 12 and 21 were examined by mass spectrometry (Fig. S1), while fractions 4, 6, 8 and 21 were studied by NMR spectroscopy (Fig. S2). All fractions were left standing in open vials for slow evaporation of the solvent. From these crystallization attempts, fractions 1, 6 and 21 gave crystals. The observation that some com­pounds were present in nearly all fractions is most likely due to the low solubility in the eluting solvent, which led to ‘smearing’ over the length of the chromatography column. The use of different solvent mixtures for elution (PE/Et_2_O, PE/THF and PE/CH_2_Cl_2_; PE is petroleum ether) increased the solubility, but did not improve the resolution of the com­pounds. This problem might have been overcome by the use of high-performance liquid chromatography (HPLC); however, this was not available to us.

The mass spectrum of fraction 1 showed the presence of **2** (*m*/*z* = 579.7), **3** (*m*/*z* = 659.6) and **4** (*m*/*z* = 737.5), with **2** as the main com­ponent. In both of fractions 10 and 12, **4** was the main com­ponent, contaminated by **2** (traces), **3** and **5** (*m*/*z* = 817.5). Finally, the mass spectrum of fraction 21 showed **5** as the main com­ponent, contaminated by **4** and traces of **6** (*m*/*z* = 895.2). The ^1^H NMR spectra of fractions 4, 6 and 8 showed different mixtures of com­pounds **3** (δ = 4.47) and **4** (δ = 4.72 and 4.43) [assignments based on Butler (2021 ▸)]. The ^1^H NMR spectrum of fraction 21 (in dimethyl sulfoxide) showed three very weak signals at δ = 5.33, 5.20 and 4.76, which might be assigned to **4** and **5** by com­parison with the mass spectra (no other NMR data in this solvent were available). The crystals obtained from fraction 1 suffered from disorder or cocrystallization effects, which could not be properly resolved. The crystals from fraction 6 also showed disorder, which could, however, be successfully modelled as cocrystallization of com­pounds **3** (*ca* 97% contribution) and **2**. Fraction 21 yielded pure crystals of com­pound **5**.

**Figure 1:**
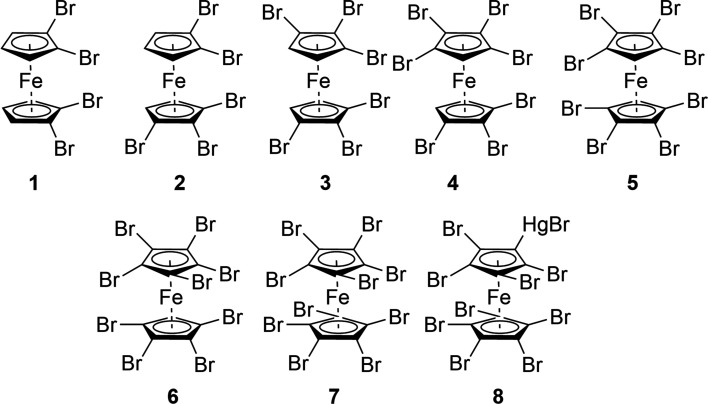
The structural formulae of com­pounds **1**–**8**.

Fig. 1 ▸ shows the structural formulae of the com­pounds discussed in this study.

We also repeated the permercuration of ferrocene according to Winter and co-workers, using Hg(O_2_CCH_3_)_2_ as the mercurating agent and di­chloro­ethane as the solvent, followed by bromination with KBr_3_. Chromatography of the crude reaction product yielded two fractions (Han *et al.*, 1994 ▸). The first contained, according to its mass spectrum (Fig. S3), a mixture of bromo­ferrocenes **5**–**7** (with com­pound **6** dominating), while the second consisted of a mixture of bromo­mercurioferrocenes [C_10_H_*n*_Br_10–*n*_HgFe] (*n* = 0–2; Fig. S4). Fig. S5 shows the ^1^H NMR spectrum of the first fraction, which apparently consists of four proton-containing substances, of which one dominates. Although we did not perform these experiments, it can be assumed that com­pounds **5**–**7** are formed by further bromination of [C_10_H_*n*_Br_10–*n*_HgFe]. Therefore, we conclude that neither the permercuration nor the bromination reactions are com­plete. Although all fractions were used for crystallization attempts, only crystals of com­pound **8** could be obtained.

### Mol­ecular structures

An intensely debated topic since the very early days of ferrocene chemistry was the question of the relative stability of the eclipsed and staggered conformers of this mol­ecule. While the very first crystal structure determination of ferrocene (Fischer & Pfab, 1952 ▸) hinted at a staggered geometry, the most recent low-temperature IR and XANES (X-ray absorption near edge structure) spectra, as well as DFT (density functional theory) calculations showed that the eclipsed conformation is the energy minimum (Bourke *et al.*, 2016 ▸; Silva *et al.*, 2014 ▸). For ferrocenes substituted on both rings, an additional conformational isomerism arises from the possibility of different relative positions of the substituents (Scheme 1). 
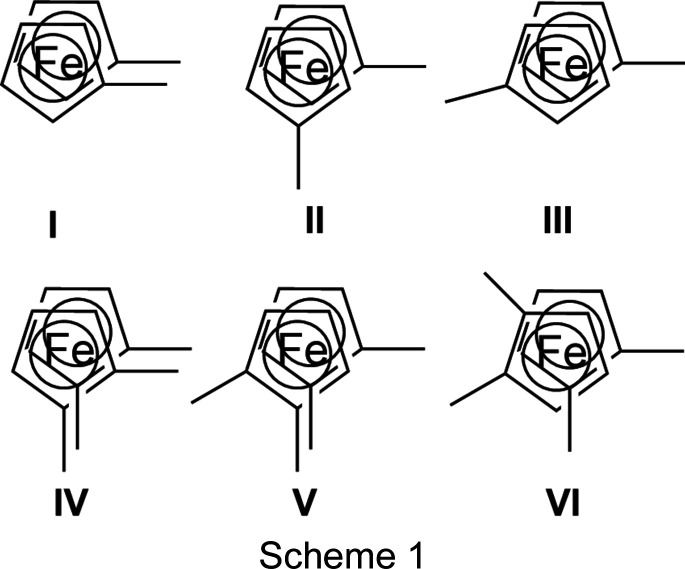


While theoretical calculations on 1,1′-di­bromo­ferrocene showed that the two *C*_2_ isomers (**II** and **III** in Scheme 1) are minimum conformations (Silva *et al.*, 2014 ▸), in the crystal structure, only the less favourable *C*_2*v*_ structure (**I** in Scheme 1) was obtained (Hnetinka *et al.*, 2004 ▸). To obtain an overview of the ‘realized’ structures, a Cambridge Structural Database (CSD; Groom *et al.*, 2016 ▸) search on ferrocenes with at least one halogen substituent on each ring was undertaken. This search delivered 40 hits, of which 14 contained only halogen substituents: all four Fd*X*_2_, two Fd*X*_3_, three Fd*X*_4_, FdCl_6_, FdBr_9_ and FdBr_10_; FdCl_2_ was determined twice and FdI_4_ exists as two positional isomers; ‘Fd’ is a common abbreviation for ferrocenes with substituents on both rings, while ‘Fc’ symbolizes ferrocenes with substituents only on one ring; strictly speaking, ‘Fc’ stands only for the [C_10_H_9_Fe] residue, while ‘Fd’ symbolizes a [(C_5_H_4_)_2_Fe] group. The four 1,1′-dihaloferrocenes have been discussed already with respect to supra­molecular inter­actions in general and halogen bonding in particular (Shimizu & Ferreira da Silva, 2018 ▸). All these com­pounds, except for FdI_2_, FdBr_9_ and FdBr_10_, showed eclipsed conformations (or nearly eclipsed in the case of FdBr_3_ and FdI_3_, with torsion angles of *ca* 14°). Within this group of eclipsed structures, most showed the apparent ‘higher energy’ conformations **I** and **IV**, respectively. Only FdBr_3_, FdI_3_ and FdI_4_ crystallized in the most stable form **VI**. Table 2 ▸ gives an overview on these structures.

**Figure 2:**
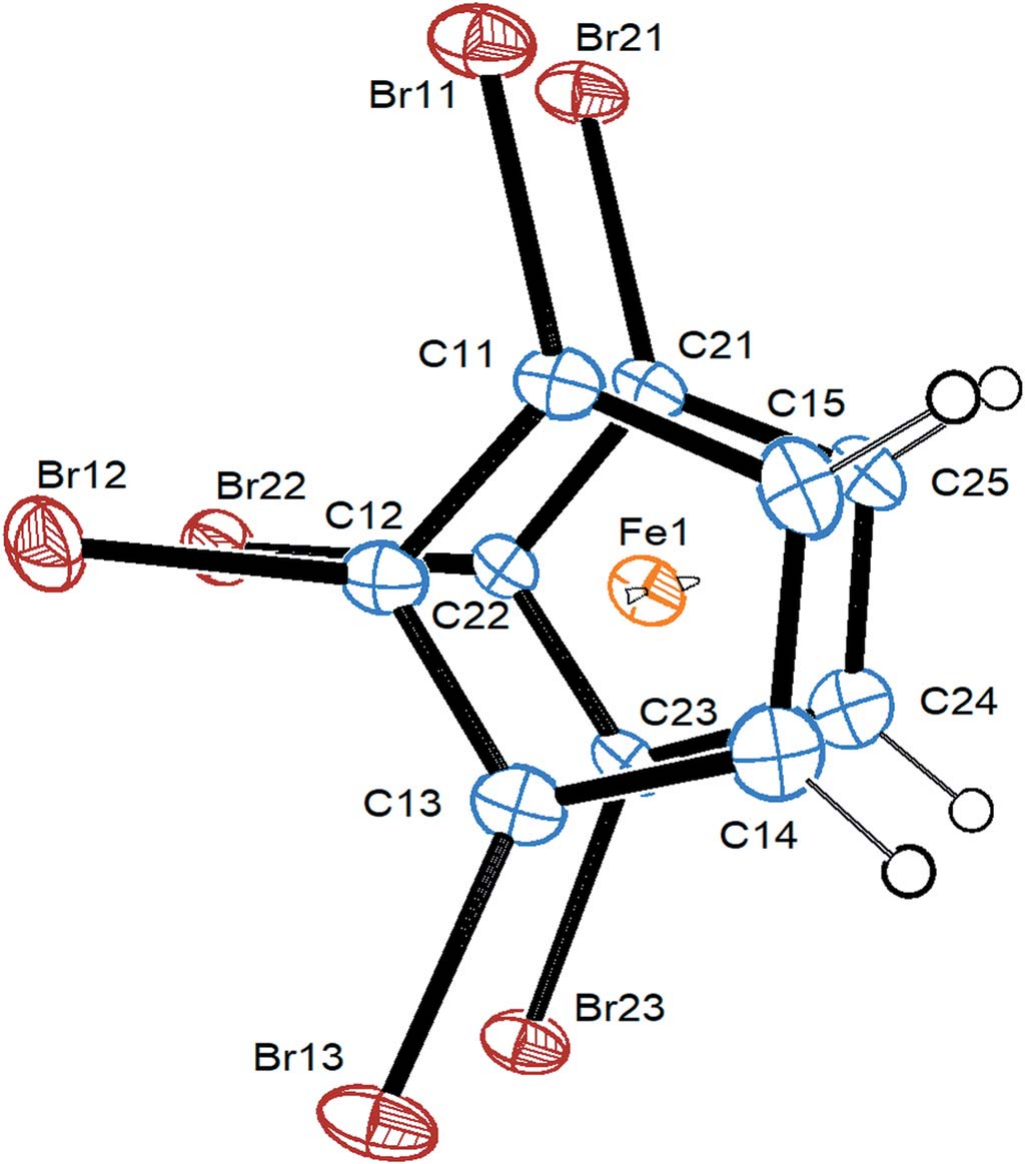
Top view of the mol­ecular structure of com­pound **3** (major orientation), with displacement ellipsoids drawn at the 30% probability level.

Compound **3** crystallizes in the triclinic space group *P* 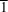 , with one mol­ecule in the asymmetric unit. Fig. 2 ▸ shows the major orientation of the disordered mol­ecule. As in most polyhaloferrocene structures (see Table 2 ▸), the cyclo­penta­dienyl (Cp) rings are nearly perfectly eclipsed, planar and parallel to each other. All Br atoms are shifted slightly to the distal side of the Cp rings with respect to the Fe atom.

**Figure 3:**
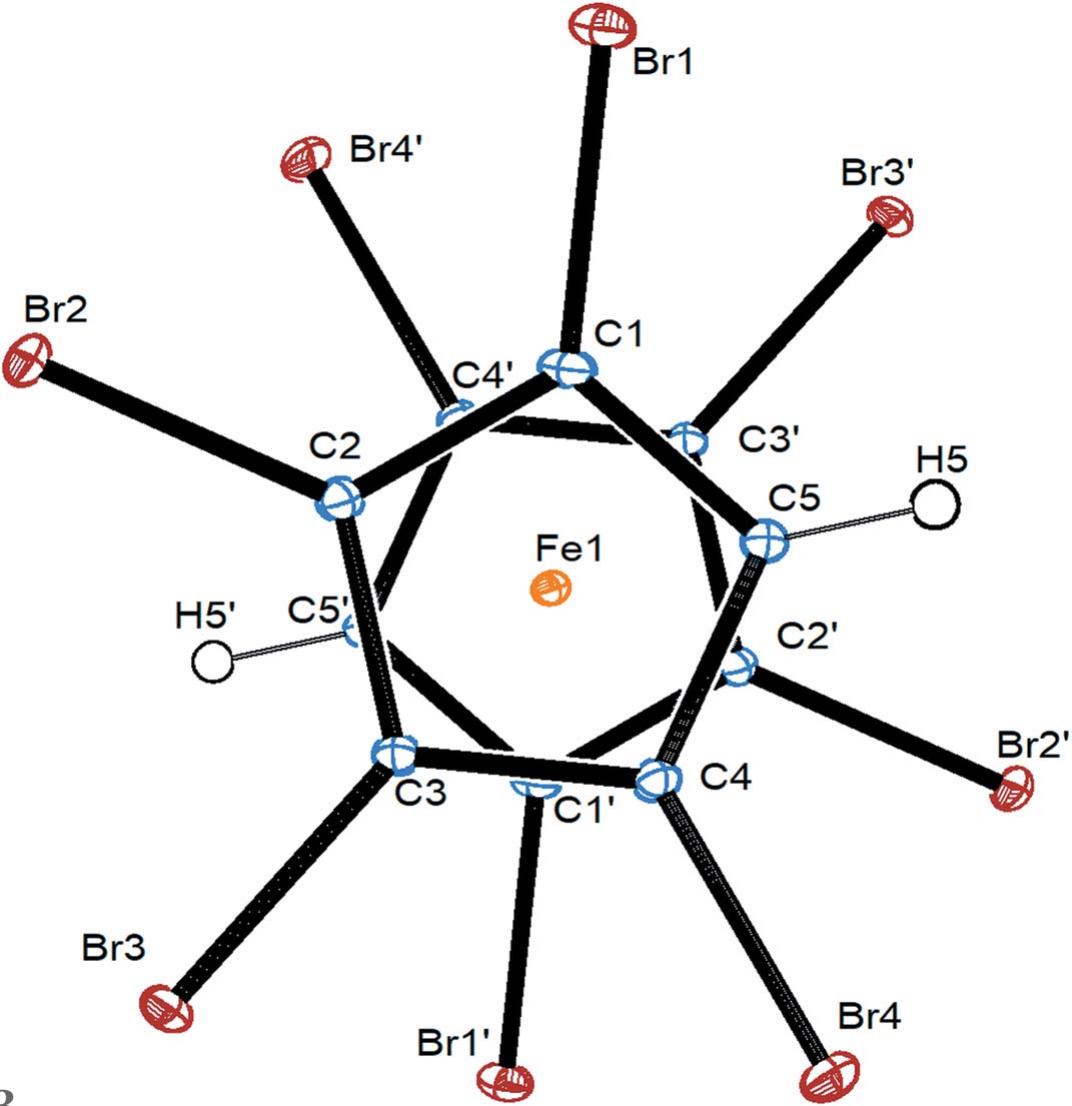
Top view of the mol­ecular structure of com­pound **5**, showing a whole mol­ecule, with displacement ellipsoids drawn at the 30% probability level.

Compound **5** also crystallizes in the triclinic space group *P* 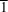 , however, as a twin with half a mol­ecule in the asymmetric unit and the Fe atom residing on an inversion centre (Fig. 3 ▸). As a consequence of this, the Cp rings are perfectly staggered, with the two C—H bonds in relative transoid positions. Both Cp rings are planar and parallel to each other and the Br atoms are all shifted to the distal sides of the Cp rings, however, to a smaller extent than in the eclipsed structures mentioned before. The iron–centroid distance (determined within *PLATON*) also seems to be more dependent on the relative orientation of the Cp rings than on the degree of bromination. Table 3 ▸ collects important geometrical parameters of several polybromo­ferrocenes from the literature, together with those of com­pounds **3**, **5** and **8**.

**Figure 4:**
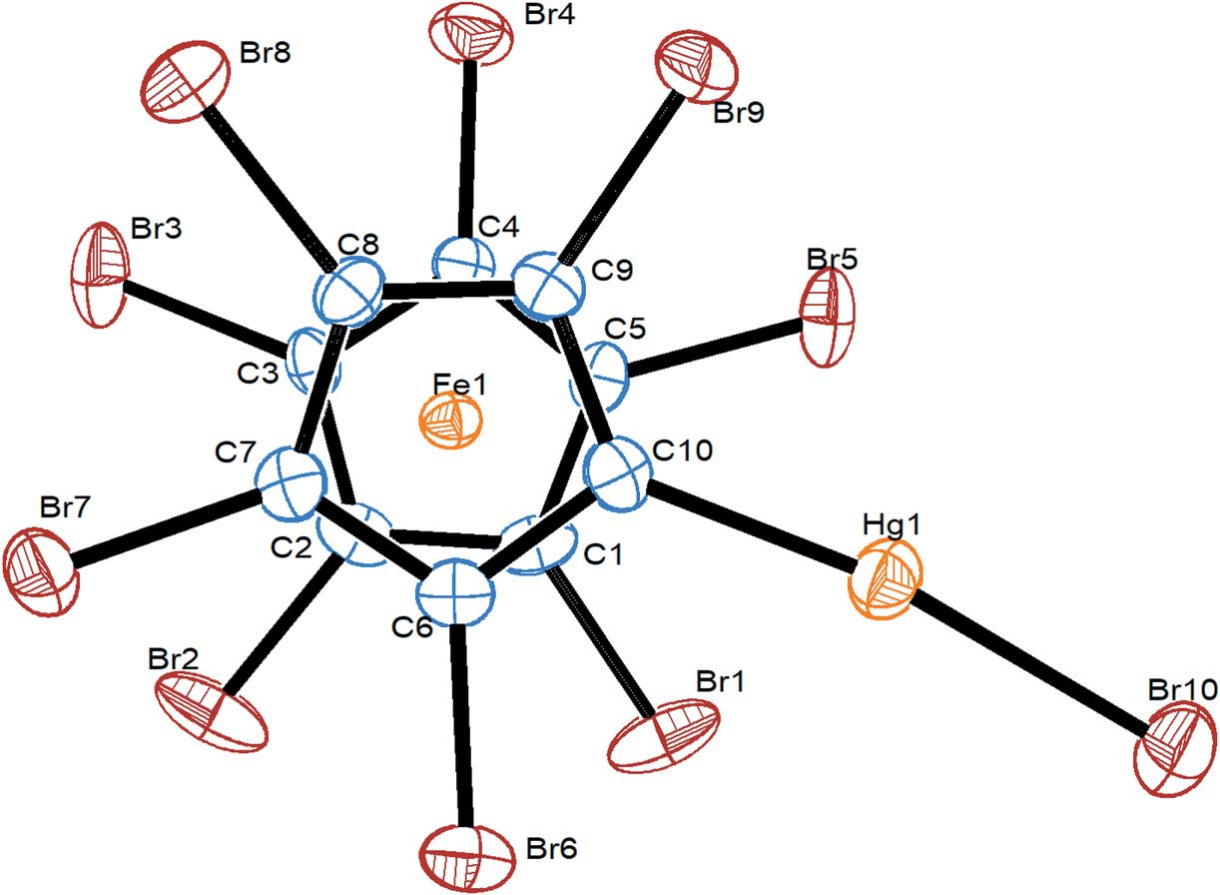
Top view of the mol­ecular structure of **8**, with displacement ellipsoids drawn at the 30% probability level.

Compound **8** crystallizes in the monoclinic space group *P*2_1_/*n*, with one mol­ecule in the asymmetric unit (Fig. 4 ▸). The C10—Hg1—Br10 bond deviates slightly from being linear [171.0 (2)°]. The Cp rings are planar and parallel to each other, while their relative orientation is staggered. The distances from Fe1 to both Cp ring centroids are identical within 1σ. Except for atoms Br5 and Hg1, which are within the Cp ring planes, all the ring substituents are shifted again to the distal sides of the Cp rings. In com­parison with the structure of the ferricenium salt **8^+^** ·AsF_6_^−^, the C—Br bonds are slightly longer, while the iron–centroid distances are significantly shorter in **8**, which is quite usual when com­paring ferrocenes with their oxidized counterparts (Rupf *et al.*, 2022 ▸).

For all three com­pounds, an analysis with *PLATON* showed no residual solvent-accessible voids (Spek, 2020 ▸).

### Hirshfeld analysis and inter­molecular contacts

To gain some insight into the inter­molecular inter­actions at work in these com­pounds, a Hirshfeld analysis was undertaken, using the program *CrystalExplorer* (Spackman *et al.*, 2021 ▸).

**Figure 5:**
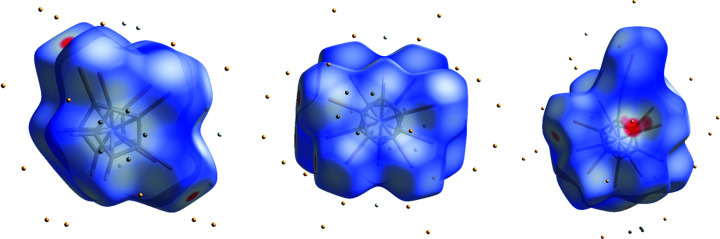
Hirshfeld surfaces of com­pounds **3** (left), **5** (middle) and **8** (right), together with the closest contact atoms. Red spots show very close contacts between atoms inside and outside the Hirshfeld surface. The Hg com­pound differs from the other two by the appearance of such a red spot over the plane of the Cp ring.

Fig. 5 ▸ shows the Hirshfeld surfaces of the three com­pounds, together with the closest contact atoms (within 3.8 Å).

**Figure 6:**
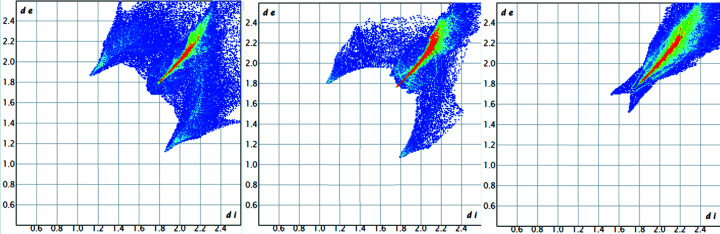
Fingerprint plots of com­pounds **3** (left), **5** (middle) and **8** (right). A red colour symbolizes a large number of points on the Hirshfeld surface at the corresponding *d*_e_/*d*_i_ pair, green inter­mediate numbers and blue small numbers.

The so-called ‘fingerprint plots’, which summarize all contacts between atoms inside and outside the Hirshfeld surface (Spackman & McKinnon, 2002 ▸; Spackman & Jayatilaka, 2009 ▸), are shown in Fig. 6 ▸. A common feature of all three plots is the occurrence of a red stripe around the main diagonal, reaching from *ca d*_e_/*d*_i_ = 1.8/1.8 to 2.2/2.2, which corresponds to a large number of Br⋯Br contacts (*d*_e_ + *d*_i_ = 3.6 to 4.4 Å; the sum of the van der Waals radii of two Br atoms is 3.70 Å).

The very different appearance of these plots is mainly due to the decreasing number of H atoms present. Table 4 ▸ and Fig. S8 of the supporting information provide a more detailed analysis, showing the different contributions of the individual element contacts.

Due to purely statistical effects (there are eight H atoms in FdBr_2_, four in **3**, two in **5** and none in **8**), the absolute numbers cannot be com­pared directly. However, it is quite obvious that the importance of C⋯H and especially H⋯H contacts decreases drastically with increasing bromine content, while the importance of Br⋯Br contacts increases in the same direction. At the same time, it appears quite inter­esting that H⋯Br contacts are very important in all com­pounds where H atoms are present.

To obtain a more detailed picture of the individual inter­actions, a *Mercury* analysis was undertaken (Macrae *et al.*, 2020 ▸)

#### Hydrogen bonds

The structures of the known polyhaloferrocenes collected in Table 2 ▸ show three different patterns of hydrogen bonding (Scheme 2): Type **A** is observed in the structures of FdF_2_, FdBr_3_, FdI_3_, FdCl_4_, FdBr_4_ and FdBr_9_. All Fd*X*_2_, as well as iodo­ferrocenes and FdBr_4_, show Type **B**, while Type **C** is seen only in the two trihaloferrocenes. 
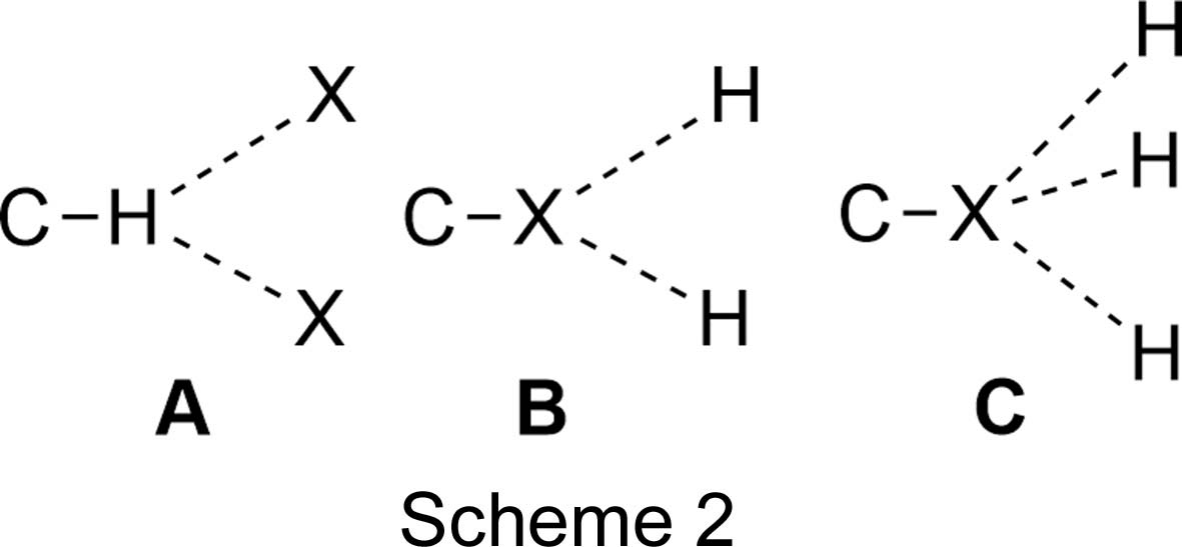


**Figure 7:**
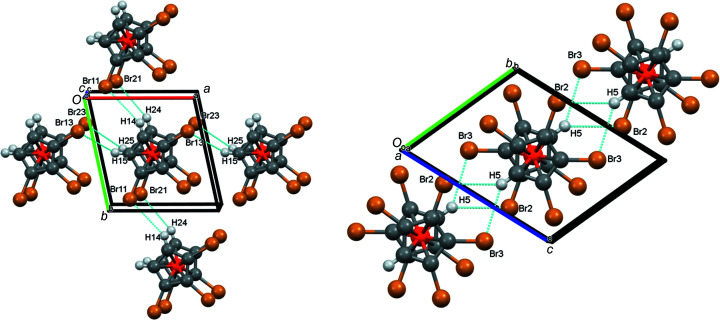
(Partial) packing plots (*Mercury*; Macrae *et al.*, 2020 ▸) of com­pounds **3** (left), viewed along *c*, and **5** (right), viewed along *a*, showing the inter­molecular hydrogen bonds. Colour codes as defined by *Mercury*: carbon dark grey, hydrogen light grey, iron orange and bromine brown; the red lines are unexpanded contacts and the cyan lines are expanded contacts.

When using the standard settings of *Mercury*, no hydrogen bonds are indicated for com­pound **3**. However, when increasing the limit by 0.2 Å, four (obviously very weak) hydrogen bonds appear [Fig. 7 ▸ (left) and Table 5 ▸].

As can be seen from Fig. 7 ▸ (left), the atom pairs H14/Br11 and H24/Br21 connect the individual mol­ecules in the *y* direction, while the pairs H15/Br13 and H25/Br23 join them in the *x* direction. Atoms Br12 and Br22 are not involved in hydrogen bonding.

In com­pound **5**, the standard settings of *Mercury* suffice to show that only atom H5 (and, of course, its inversion-symmetry-generated counterpart H5′) engages in a symmetrical bifurcated hydrogen bond (Type **A** in Scheme 2) with atoms Br2 and Br3 [Fig. 7 ▸ (right) and Table 5 ▸]. As the figure shows, these inter­actions join individual mol­ecules in the *y* direction.

#### Halogen bonding and other Br⋯Br inter­actions

In the discussion of halogen bonding, a distinction is usually made between XB Type I and XB Type II. According to the IUPAC and IUCr classifications, Type I contacts are geometry based, arising from close-packing requirements, while Type II arise from inter­actions between an electron-rich region on one halogen atom and an electron-deficient region on the other. The distinction can be made on the basis of the angles Θ1 and Θ2, which occur at halogen atoms *X* and *X*′ of *R*—*X*⋯*X*′—*R*′, and their difference (Scheme 3). 
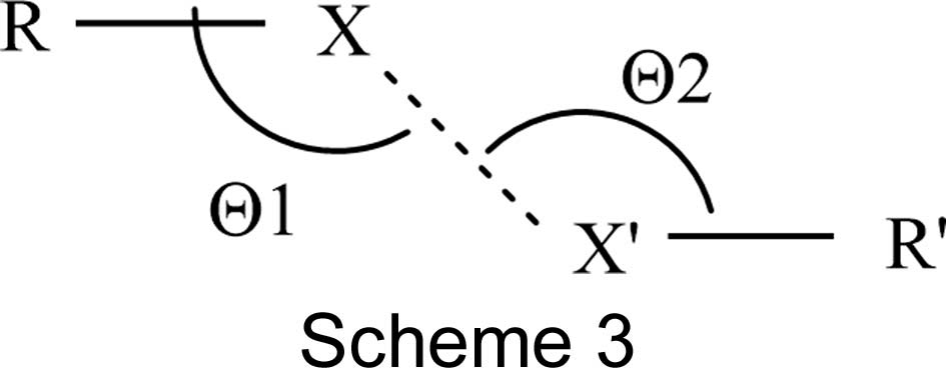


Usually it is assumed that for 0 < |Θ1 – Θ2| < 15°, a Type I contact is formed, while for Type II contacts, 30 < |Θ1 – Θ2| < 105° is found, and only the latter are regarded as real halogen bonds (Cavallo *et al.*, 2016 ▸). In a more recent article, a distinction into Types I–IV was suggested, but was still based on these angles (Ibrahim *et al.*, 2022 ▸): Type I: 90 < Θ1 ≃ Θ2 < 180°; Type II: Θ1 = 180° and Θ2 = 90°; Type III: Θ1 ≃ Θ2 = 180°; Type IV: Θ1 ≃ Θ2 = 90° (obviously, Types III and IV are only extrema of the more general Type I). The forces behind these attractions are either van der Waals (Type I), electrostatic (Type II) or dispersion (Types III and IV) forces. It was further found that ‘the Type I inter­actions were more frequent at the shortest distances’ (Cavallo *et al.*, 2016 ▸). Table 6 ▸ collects the structural parameters of com­pounds **3**, **5** and **8**, while Figs. S9–S11 show *Mercury* representations of these inter­actions.

All the listed Br⋯Br contacts in Table 6 ▸ are well below the sum of the van der Waals radii (3.70 Å; Bondi, 1964 ▸). When using the |Θ1 – Θ2| criterion, most inter­actions classified as Type II are also ‘real’ halogen bonds. To look at the structural consequences of this halogen bonding, a visualization of the packing plots should be helpful.

**Figure 8:**
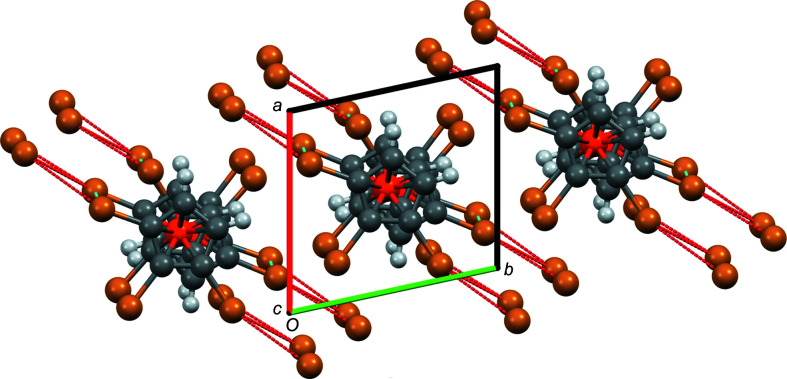
Packing plot of com­pound **3**, viewed along *c*. Colour codes as defined by *Mercury*: carbon dark grey, hydrogen light grey, iron orange and bromine brown; the red lines are unexpanded contacts and the cyan lines are expanded contacts.

**Figure 9:**
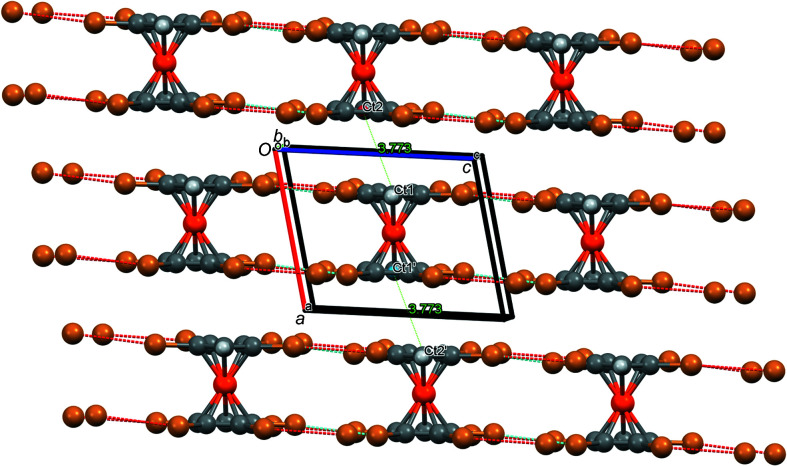
Packing plot of com­pound **5**, viewed along *b*. Colour codes as defined by *Mercury*: carbon dark grey, hydrogen light grey, iron orange and bromine brown; the red lines are unexpanded contacts and the cyan lines are expanded contacts.

Fig. 8 ▸ shows how the Br⋯Br inter­actions join the individual mol­ecules of com­pound **3** in the direction of the *xy* diagonal (*b*–*a* vector). It can also be seen that there are two *intra*mol­ecular Br⋯Br contacts of Type IV, emphasized in italic in Table 5 ▸. Two Br atoms (Br12 and Br22) are not involved in Br⋯Br inter­actions. Fig. 9 ▸ shows that in com­pound **5** the Br⋯Br contacts join the individual mol­ecules in the *z* direction. All eight Br atoms are involved in Br⋯Br inter­actions. In addition, there is also some π–π stacking in the *x* direction; the centroids of two adjacent Cp rings are only 3.773 Å apart, while the ring planes have an inter­planar distance of 3.507 Å (corresponding to an angle of 21.6° between the Ct—Ct′ vector and the plane normal).

**Figure 10:**
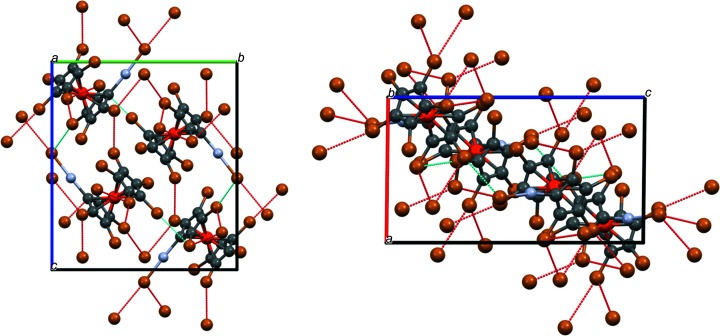
Packing plots of com­pound **8**, viewed along *a* (left) and along *b* (right). Colour codes as defined by *Mercury*: carbon dark grey, hydrogen light grey, iron orange and bromine brown; the red lines are unexpanded contacts and the cyan lines are expanded contacts.

In bromo­mercurio com­pound **8**, matters are a bit more com­plicated. Fig. 10 ▸ shows that Br⋯Br contacts join the individual mol­ecules in all directions. All Br atoms, except for Br2, Br5 and Br8, are involved in Br⋯Br contacts.

**Figure 11:**
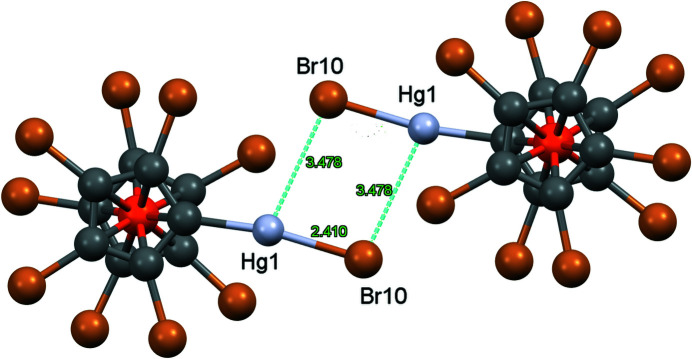
The Hg_2_Br_2_ ring in com­pound **8**. Colour codes as defined by *Mercury*: carbon dark grey, hydrogen light grey, iron orange and bromine brown; the red lines are unexpanded contacts and the cyan lines are expanded contacts. Generic atom labels without symmetry codes ahve been used.

**Figure 12:**
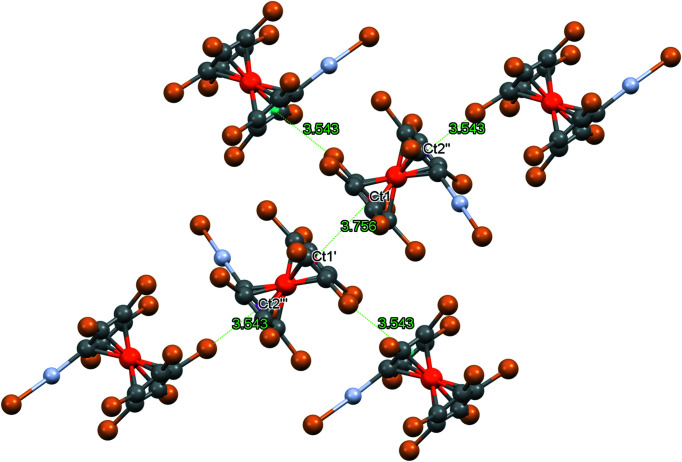
Partial packing diagram of com­pound **8**, showing the Br⋯π and π–π contacts (Å). Colour codes as defined by *Mercury*: carbon dark grey, hydrogen light grey, iron orange, mercury blue and bromine brown; the red lines are unexpanded contacts and the cyan lines are expanded contacts. Ct1′/Ct1′′ and Ct2′/Ct2′′ are the centroids of inversion-related cyclo­penta­dienyl rings, with one Fe atom between Ct1′ and Ct2′, and another between Ct1′′ and Ct2′′.

But there are more inter­actions involving Br atoms. First there are Hg⋯Br contacts, shown in Fig. 11 ▸. The Hg1⋯Hg1 distance is 4.4944 (6) Å and therefore any mercurophilic inter­actions (Schmidbaur & Schier, 2015 ▸) can be excluded. In the crystal of the ferricenium com­plex **8** ^+^·AsF_6_^−^, there is also a Hg_2_Br_2_ ring with significantly shortened inter­molecular Hg⋯Br contacts of 3.061 Å and a Hg⋯Hg distance of 3.993 Å (Rupf *et al.*, 2022 ▸). Furthermore, there are Br⋯π contacts of 3.543 Å to a close Cp ring, in addition to a weak π–π inter­action between two Cp rings (Fig. 12 ▸); π–π stacking occurs between two inversion-related C_5_Br_5_ rings. Since the difference between the Ct—Ct′ distance of 3.756 Å and the perpendicular distance between the Cp ring planes (3.690 Å) is rather small (corresponding to an angle of 10.8° between the Ct—Ct′ vector and the plane normal), it can be regarded as a ‘true’ π–π inter­action (though rather weak).

A similar Br⋯π inter­action was found in the structure of FdBr_2_; however, it was, with a Br⋯centroid distance of 3.824 Å, substanti­ally weaker (Shimizu & Ferreira da Silva, 2018 ▸).

#### Co-operativity between H⋯Br and Br⋯Br contacts

**Figure 13:**
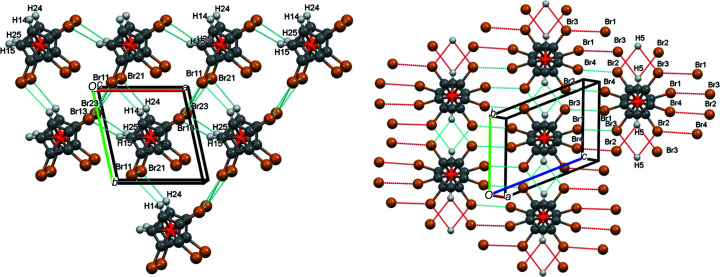
Co-operativity of hydrogen and halogen bonding in com­pounds **3** and **5**. Colour codes as defined by *Mercury*: carbon dark grey, hydrogen light grey, iron orange and bromine brown; the red lines are unexpanded contacts and the cyan lines are expanded contacts.

The importance of co-operativity in noncovalent inter­actions in general (Mahadevi & Sastry, 2016 ▸) and for the inter­play of halogen and hydrogen bonds (Decato *et al.*, 2021 ▸; Portela & Fernández, 2021 ▸) in particular has been recognized in recent years and has been modelled by DFT calculations. This inter­play has also been discussed for the 1,1′-dihaloferrocenes (Shimizu & Ferreira da Silva, 2018 ▸). In the preceding sections, we have discussed the individual contributions in com­pounds **3** and **5**, and a look at Fig. 13 ▸ (and Tables 4 ▸ and 5 ▸) shows that also in these com­pounds HB and XB work together on the same halogen atoms.

#### Energetics of the inter­molecular inter­actions found in com­pounds 3, 5 and 8

**Figure 14:**
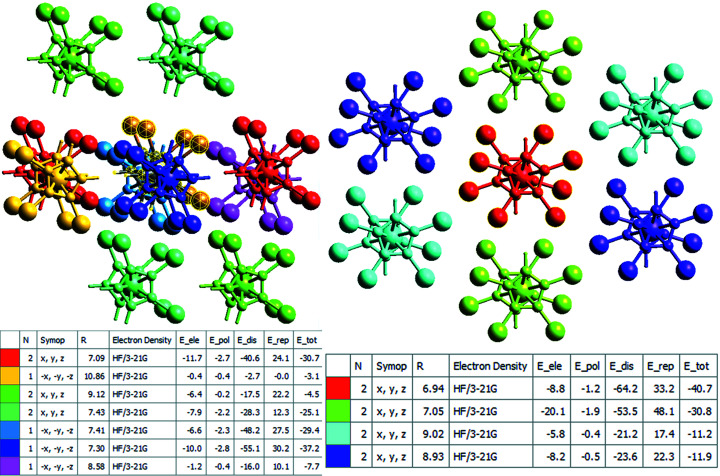
Inter­action energies (HF/3-21G) for com­pounds **3** (left) and **5** (right) (standard program settings). The colour codes in the images refer to the tables below them.

The program *CrystalExplorer* allows for the calculation of inter­action energies using the DFT program *TONTO* at the HF/3-21G level (Mackenzie *et al.*, 2017 ▸). Fig. 14 ▸ shows the results of calculations for com­pounds **3** and **5** (apparently, due to the presence of Hg, the program cannot calculate wavefunctions for com­pound **8**).

**Figure 15:**
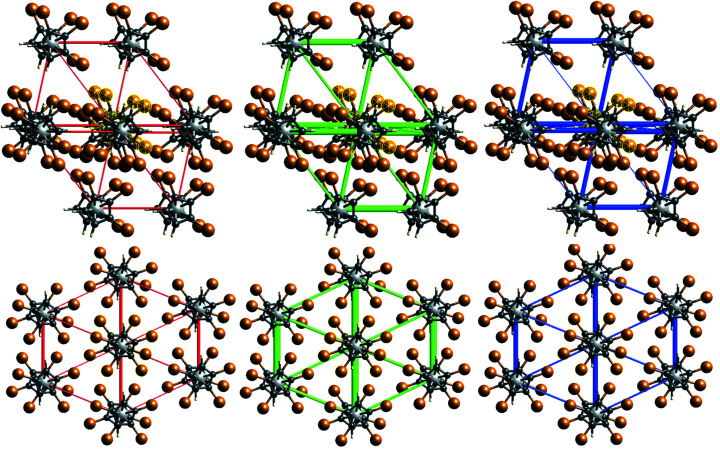
Energy frameworks (Coulombic energy in red, dispersion energy in green and total energy in blue) for com­pounds **3** (top) and **5** (bottom).

Inspection of the numerical values shows that the total inter­action energies are stronger for com­pound **3**. This is apparently due to the larger repulsion terms for **5**, because both the largest dispersion and the largest electrostatic terms are found in com­pound **5**. Another graphical representation (‘energy frameworks’) of the individual contributions can be seen in Fig. 15 ▸.

#### Comparison with halogen bonding in other haloferrocenes Fd*X*_*n*_ with *X* ≠ Br and *n* > 2

At this point, it seems worthwhile to look at the occurrence of halogen bonding in the other polyhaloferrocenes mentioned in Table 2 ▸. As mentioned already, this study has been performed for the 1,1′-dihaloferrocenes before, and therefore these structures will not be considered here again. Instead, the structure of the homoannularly substituted penta­bromo­ferrocene (FcBr_5_; Sünkel & Bernhartzeder, 2011 ▸) is included (Table 7 ▸). All the listed *X*⋯*X* contacts are below the sum of the van der Waals radii and of Type II except for the chloro com­pound (0.004 Å longer than this sum and Type I). This result (the increasing importance of *X*⋯*X* contacts when going from *X* = Cl to *X* = I) parallels the observations in the Fd*X*_2_ sytems. In addition to the *X*⋯*X* inter­actions, C—H⋯*X* hydrogen bonds are important for all com­pounds, especially the chloro com­pound. π–π inter­actions are very strong for FdI_4_ (virtually no displacement of the Cp rings of different mol­ecules), while in FdCl_4_, the shift between the perpendicular projection of one centroid to the centroid of a neighbouring mol­ecule is quite substantial. In FdI_3_, C—H⋯π inter­actions seem to be of some importance, while in FcBr_5_, a weak C—Br⋯π inter­action can be observed.

## Conclusion

Both stepwise deprotonation/electrophilic bromination starting from 1,1′,2,2′-tetra­bromo­ferrocene and permercuration/bromination of ferrocene lead to mixtures of polybrominated ferrocenes. However, by a combination of chromatography and recrystallization, it was possible to obtain crystals of hexa- and octa­bromo­ferrocene, as well as of nona­bromo(bromo­mer­cur­io)ferrocene. Hexa­bromo­ferrocene shows an eclipsed conformation of the Cp rings, as was also found for the already known structures of 1,1′-di­bromo- and 1,1′,2,2′-tetra­bromo­ferrocene. Ferrocenes with a higher bromine content apparently prefer a staggered conformation, as was observed before for nona- and deca­bromo­ferrocene. All three title com­pounds show a com­bination of halogen bonding with either hydrogen bonding or π–π inter­actions. Dispersion inter­actions appear to be stronger than electrostatic inter­actions.

## Supplementary Material

Crystal structure: contains datablock(s) compd_3, compd_5, compd_8, global. DOI: 10.1107/S205322962200955X/qw3001sup1.cif

Structure factors: contains datablock(s) compd_3. DOI: 10.1107/S205322962200955X/qw3001compd_3sup2.hkl

Structure factors: contains datablock(s) compd_5. DOI: 10.1107/S205322962200955X/qw3001compd_5sup3.hkl

Structure factors: contains datablock(s) compd_8. DOI: 10.1107/S205322962200955X/qw3001compd_8sup4.hkl

Additional figures. DOI: 10.1107/S205322962200955X/qw3001sup5.pdf

CCDC references: 2210089, 2210088, 2210087

## Figures and Tables

**Table 1:** Experimental details Experiments were carried out with Mo *K*α radiation.

	**3**	**5**	**8**
Crystal data			
Chemical formula	[Fe(C_5_H_2_Br_3_)_2_]	[Fe(C_5_HBr_4_)_2_]	[FeHgBr(C_5_Br_4_)(C_5_Br_5_)]
*M*_r_	656.69	817.25	1175.64
Crystal system, space group	Triclinic, *P* 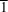	Triclinic, *P* 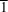	Monoclinic, *P*2_1_/*n*
Temperature (K)	153	103	295
*a*, *b*, *c* (Å)	7.0903 (3), 7.4318 (5), 13.8071 (5)	6.9395 (2), 7.0548 (2), 8.9271 (3)	8.9784 (3), 14.0971 (4), 15.8485 (4)
α, β, γ (°)	88.745 (4), 84.993 (3), 77.728 (4)	67.577 (1), 76.160 (1), 86.461 (1)	90, 90.689 (1), 90
*V* (Å^3^)	708.21 (6)	392.06 (2)	2005.79 (10)
*Z*	2	1	4
μ (mm^−1^)	17.86	21.33	28.28
Crystal size (mm)	0.49 × 0.15 × 0.05	0.03 × 0.01 × 0.01	0.06 × 0.02 × 0.02

Data collection			
Diffractometer	Agilent XCalibur 2	Bruker D8 Venture	Bruker D8 Venture
Absorption correction	Multi-scan (*CrysAlis PRO*; Agilent, 2014 ▸)	Multi-scan (*TWINABS*; Bruker, 2012 ▸)	Multi-scan (*SADABS*; Krause *et al.*, 2015 ▸)
*T*_min_, *T*_max_	0.434, 1.000	0.180, 0.344	0.193, 0.332
No. of measured, independent and observed [*I* > 2σ(*I*)] reflections	9297, 3234, 2496	3772, 3772, 3107	33353, 4098, 3154
*R*_int_	0.041	–	0.050
(sin θ/λ)_max_ (Å^−1^)	0.649	0.832	0.625

Refinement			
*R*[*F* ^2^ > 2σ(*F* ^2^)], *wR*(*F* ^2^), *S*	0.043, 0.090, 1.09	0.037, 0.076, 1.06	0.036, 0.092, 1.06
No. of reflections	3234	3772	4098
No. of parameters	162	89	199
No. of restraints	2	0	0
H-atom treatment	H-atom parameters constrained	H-atom parameters constrained	–
Δρ_max_, Δρ_min_ (e Å^−3^)	2.31, −0.97	1.32, −1.31	1.63, −1.24

Computer programs: *CrysAlis PRO* (Agilent, 2014 ▸), *APEX2* (Bruker, 2012 ▸), *SAINT* (Bruker, 2011 ▸), *SHELXT2014* (Sheldrick, 2015*a* ▸) and *SHELXL2018* (Sheldrick, 2015*b* ▸).

**Table 2:** Overview of the CSD structures of polyhaloferrocenes substituted on both rings

Chemical formula	Abbreviation in this text	Refcode in the CSD	Conformation	Reference
C_10_H_8_F_2_Fe	FdF_2_	RACROF	Eclipsed, **I**	Inkpen *et al.* (2015 ▸)
C_10_H_8_Cl_2_Fe	FdCl_2_	DUTSUH, DUTSUH01	Eclipsed, **I**	Bryan & Leadbetter (1986 ▸); Inkpen *et al.* (2015 ▸)
C_10_H_8_Br_2_Fe	FdBr_2_	BIPDOU	Eclipsed, **I**	Hnetinka *et al.* (2004 ▸)
C_10_H_8_I_2_Fe	FdI_2_	KOPFAY	Staggered	Roemer & Nijhuis (2014 ▸)
C_10_H_7_Br_3_Fe	FdBr_3_	UTOBIR	Nearly eclipsed, **VI**	Butler *et al.* (2021 ▸)
C_10_H_7_I_3_Fe	FdI_3_	EZAWUA	Nearly eclipsed, **VI**	Evans *et al.* (2021 ▸)
C_10_H_6_Cl_4_Fe	FdCl_4_	CEVBEK	Eclipsed, **IV**	Sato *et al.* (1984 ▸)
C_10_H_6_Br_4_Fe	FdBr_4_	UTOBUD	Eclipsed, **IV**	Butler *et al.* (2021 ▸)
C_10_H_6_I_4_Fe-	FdI_4_	EZAWOU	Eclipsed, **VI**	Evans *et al.* (2021 ▸)
C_10_H_4_Cl_6_Fe	FdCl_6_	DUTSUG	No data in CSD	Bryan & Leadbetter (1986 ▸)
C_10_HBr_9_Fe	FdBr_9_	FEFZAV	Staggered	Rupf *et al.* (2022 ▸)
C_10_Br_10_Fe	FdBr_10_	FEFYUO	staggered	Rupf *et al.* (2022 ▸)

**Table 3:** Important geometrical parameters of com­pounds **3**, **5** and **8** in com­parison with literature data for closely related com­pounds FdBr_2_ is 1,1′-di­bromo­ferrocene; ‘Ct’ is the abbreviation for the ‘centroid’ of the Cp rings, as calculated by the corresponding feature in *PLATON* (Spek, 2020 ▸); δ (Br—Cp) is the distance of the Br atoms from the Cp plane.

Compound	C—Br (Å)	Fe—C (Å)	Fe—Ct (Å)	Ct—Fe—Ct′ (°)	Br—Ct—Ct′—Br′ (°)	δ (Br—Cp) (Å)	Reference
FdBr_2_	1.882 (4)/1.866 (4)	2.035 (4)–2.054 (4)	1.6500 (5)/1.6483 (5)	177.71 (4)	1.55 (1)	0.137 (6)/0.082 (6)	*A*
**1**	1.873 (2)–1.877 (2)	2.036 (2)–2.052 (2)	1.6482 (8)	177.75 (6)	1.59 (8)	0.130 (1)–0.149 (1))	*B*
**3**	1.862 (7)–1.881 (6)	2.033 (6)–2.064 (6)	1.653 (3)/1.654 (3)	176.3 (2)	2.09–2.38	0.123 (1)–0.168 (1)	This work
**5**	1.865 (3)–1.874 (3)	2.036 (6)–2.056 (3)	1.6449 (16)	180	35.9–36.2	0.037 (1)–0.096 (1)	This work
**6**	1.861 (10)–1.888 (11)	2.02 (1)–2.06 (1)	1.637 (1)/1.642 (1)	178.5 (3)	33.4 (5)	0.005 (1)–0.146 (1)	*C*
**7**	1.863 (4)–1.874 (4)	2.041 (4)–2.049 (4)	1.645 (2)	180	33.8 (2)	0.085 (1)–0.142 (1)	*C*
**8**	1.852 (9)–1.880 (8)	2.024 (8)–2.049 (8)	1.641 (4)/1.644 (4)	178.4 (7)	30.5 (1)–31.6 (1)	0.004 (14)–0.142 (13)	This work
**8** ^+^·AsF_6_	1.845 (8)–1.865 (8)	2.066 (8)–2.116 (8)	1.703 (4)/1.708 (4)	178.9 (5)	32.5 (4)	−0.056 (1)–0.062 (1)	*C*

References: (*A*) Hnetinka *et al.* (2004 ▸); (*B*) Butler *et al.* (2021 ▸); (*C*) Rupf *et al.* (2022 ▸).

**Table 4:** Individual contributions (%) of the different inter­actions present in the crystal structures of FdBr_2_, **3**, **5** and **8**

Compound	C⋯H	C⋯Br	C⋯C	H⋯H	H⋯Br	Br⋯Br	Hg⋯Br
FdBr_2_*	17.1	3.7	0	37.3	39.6	2.3	–
1**	6.2	1.6	6.0	14.2	52.4	19.6	–
**3**	3.3	4.0	5.9	0.8	48.0	38.2	–
**5**	1.2	6.8	5.9	0.9	20.9	64.3	
**8**	–	10.7	3.5	–	–	77.7	8.2

Notes: (*) taken from Shimizu & Ferreira da Silva (2018 ▸); (**) calculated from the downloaded CIF file, available from the CCDC as CSD refcode UTOBUB.

**Table 5:** Hydrogen-bond parameters (Å, °) in com­pounds **3** and **5** Calculated with *SHELXL2018* (Sheldrick, 2015*b* ▸) command HTAB.

	*D*—H⋯*A*	*D*—H	H⋯*A*	*D*⋯*A*	*D*—H⋯*A*
**3**	C24—H24⋯Br21^i^	0.95	3.10	3.965 (9)	151.8
	C25—H25⋯Br23^ii^	0.95	3.13	3.874	136.5
	C14—H14⋯Br11^i^	0.95	3.20	4.046 (9)	149.8
	C15—H15⋯Br13^ii^	0.95	3.24	3.927 (8)	131.8
**5**	C5—H5⋯Br2^i^	0.95	2.985	3.786	142.93
	C5—H5⋯Br3^i^	0.95	3.015	3.809	141.91

Symmetry codes: (i) *x*, *y* − 1, *z*; (ii) *x* − 1, *y*, *z*.

**Table 6:** Characteristics of the Br⋯Br inter­actions found in com­pounds **1**, **3**, **5**, **8** and FdBr_2_

Compound	*R*—Br⋯Br′—*R*′	Br⋯Br (Å)	Θ1 (°)	Θ2 (°)	|Θ1 − Θ2| (°)	XB Type
FdBr_2_*	C1—Br1⋯Br2—C6	3.586	89.7	153.1	63.2	II
1**	C1—Br1⋯Br2—C2	3.564	145.2	156.4	11.2	I
**3**	C13—Br13⋯Br11—C11	3.617	135.2	153.8	18.6	Quasi-Type I/Type II
	C23—Br23⋯Br11—C11	3.582	137.3	143.3	6.0	I
	C23—Br23⋯Br21—C21	3.594	146.9	141.8	5.1	I
	*C13—Br13⋯Br23—C23*	*3.656*	*84.7*	*85.1*	*0.4*	IV
	*C11—Br11⋯Br21—C21*	*3.657*	*85.2*	*84.4*	*0.8*	IV
**5**	C1—Br1⋯Br3—C3	3.518	160.8	124.8	36.0	II
	C2—Br2⋯Br4—C4	3.538	128.9	163.8	34.9	II
**8**	C1—Br1⋯Br9—C9	3.545	154.8	117.4	37.4	II
	C4—Br4⋯Br6—C6	3.634	117.7	113.1	4.6	I
	C4—Br4⋯Br7—C7	3.657	171.4	112.5	58.9	II
	C3—Br3⋯Br6—C6	3.521	174.1	65.9	108.2	II
	C7—Br7⋯Br10—Hg1	3.658	167.0	97.9	69.1	II
	C9—Br9⋯Br10—Hg1	3.642	109.4	163.4	54.0	II

Notes: (*) taken from Shimizu & Ferreira da Silva (2018 ▸); (**) calculated from the downloaded CIF file, available from the CCDC as CSD refcode UTOBUB.

**Table 7:** Characteristics of the *X*⋯*X* inter­actions in FdCl_4_, FdI_3_, FdI_4_ and FcBr_5_

	*R*—*X*⋯*X*′—*R*′	*X*⋯*X* (Å)	Θ1 (°)	Θ2 (°)	|Θ1 − Θ2| (°)	XB Type
FdI_3_	C1—I1⋯Br2—C2	3.74p	90.5	172.6	82.1	II
	C1—I1⋯I3—C6	3.728	174.4	115.1	59.3	II
FdI_4_	C1—I1⋯I3—C6	3.679	165.1	79.9	85.2	II
	C2—I2⋯I12—C12	3.933	159.6	97.1	62.5	II
	C6—I3⋯I13—C16	3.756	99.4	169.3	69.9	II
	C7—I4⋯I12—C12	3.823	83.0	165.8	82.8	II
	C11—I11⋯I13—C16	3.823	163.0	70.3	92.7	II
FdCl_4_	C11—Cl1⋯Cl2—C21	3.504	165.5	161.5	4.0	I
FcBr_5_	C2*A*—Br2*A*⋯Br3*B*—C3*B*	3.352	137.3	168.4	31.1	II
	C2*B*—Br2*B*⋯Br3*B*—C3*B*	3.656	164.7	123.2	41.5	II
