# Supplementary material for: Isolation and crystal and mol­ecular structures of [(C5H2Br3)2Fe], [(C5HBr4)2Fe] and [(C5Br5)(C5Br4HgBr)Fe]
Source: Acta Crystallogr C Struct Chem. 2022 Oct 13;78(Pt 11):578–90. doi: 10.1107/S205322962200955X (PMC9635591; doi:10.1107/S205322962200955X)
Supplement: Supplementary file 5 [file c-78-00578-sup5.pdf]

STRUCTURAL CHEMISTRY

Volume 78 (2022)

Supporting information for article:

Isolation and crystal and molecular structures of $[(C_5H_2Br_3)_2Fe]$, $[(C_5HBr_4)_2Fe]$ and $[(C_5Br_5)(C_5Br_4HgBr)Fe]$

Tobias Blockhaus and Karlheinz Sünkel

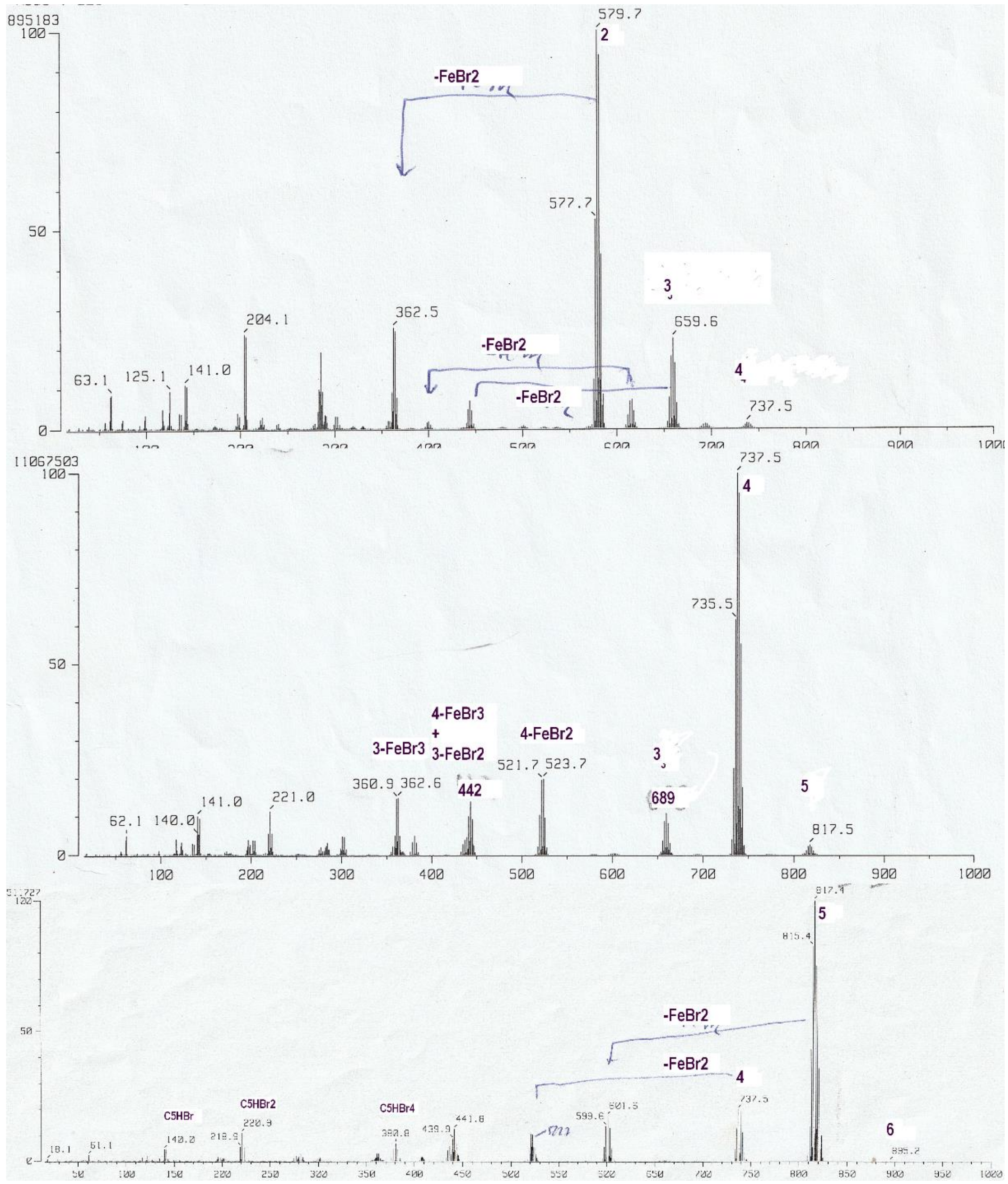

Figure S1 EI Mass spectra of fractions 1, 10 and 21 from the reaction of compd. **1** with 10 eq. LiTMP and $C_2H_2Br_4$. Assignment of molecular ions and fragmentation ions was confirmed by independent calculations of the corresponding isotope patterns.

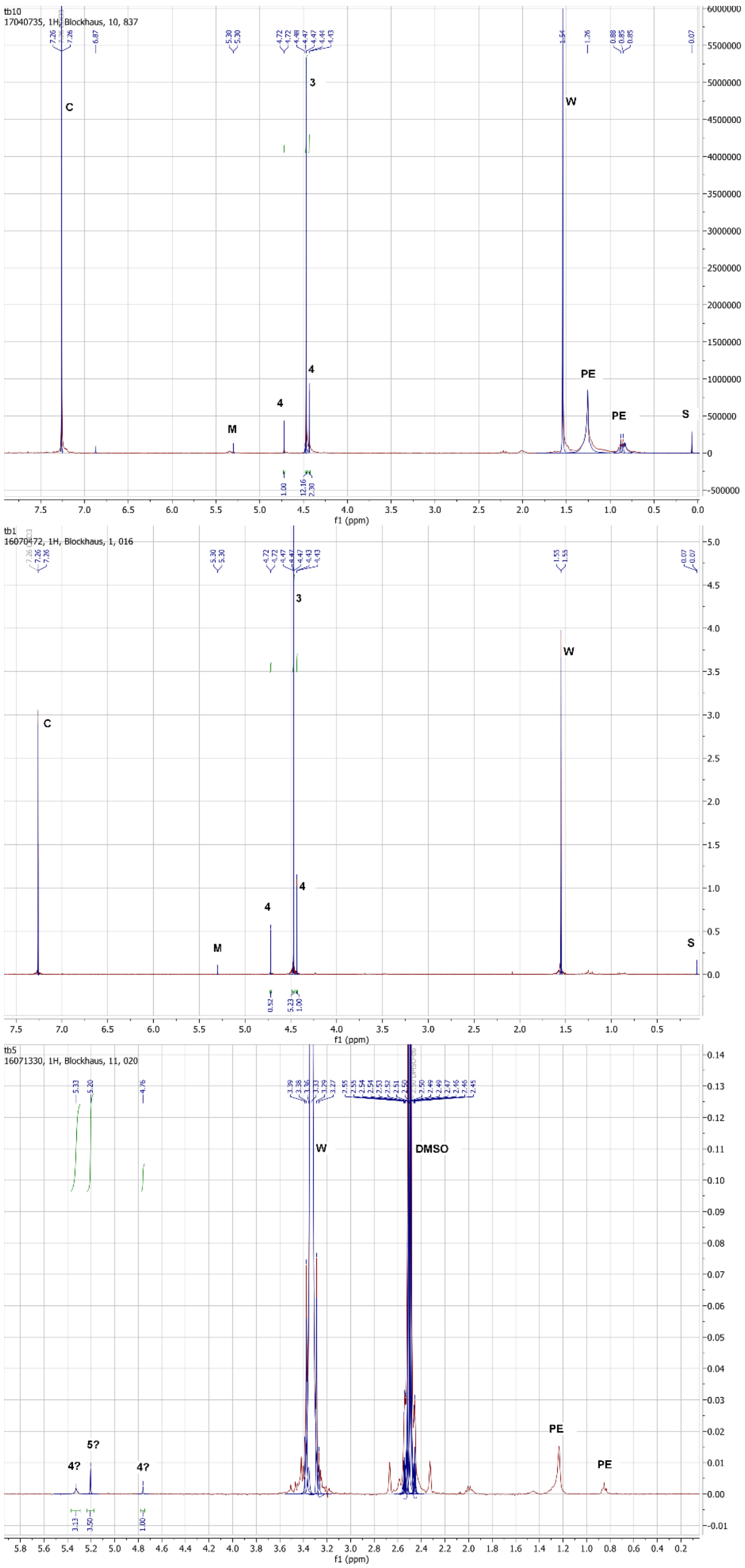

Figure S2 ^{1}H-NMR spectra ($CDCl_3$) of fractions 6, 8 and 21 from the reaction of compd. **1** with 10 eq. LiTMP and $C_2H_2Br_4$. Assignment of the individual signals based on comparison with literature

values. Impurities: C= $CHCl_3$; W= water; PE = petroleum ether; S= silicon grease; M= methylene chloride

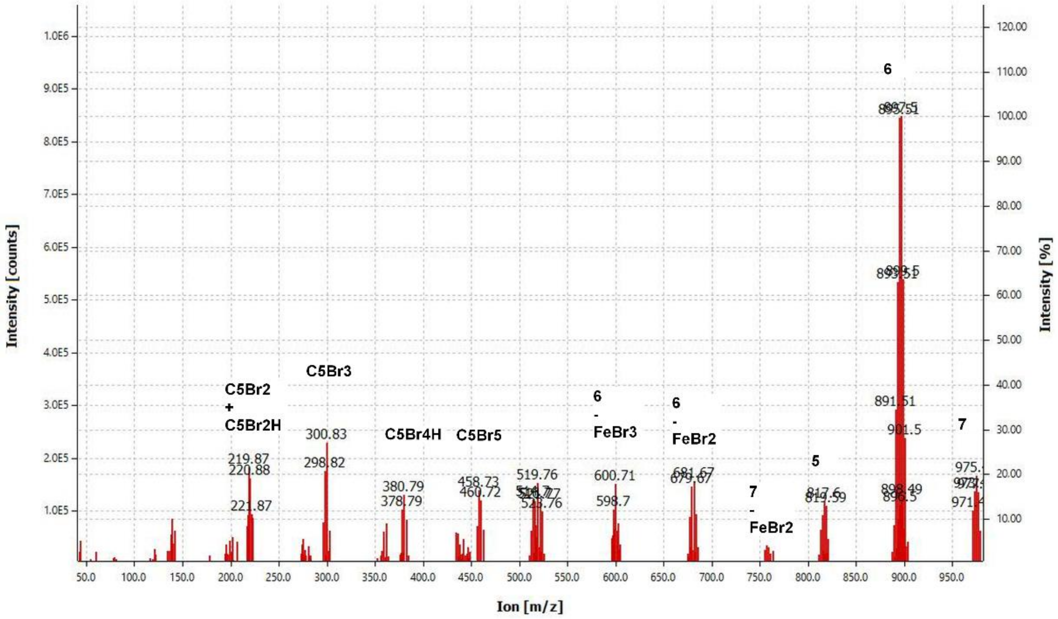

Figure S3 EI Mass spectrum of the first fraction from the reaction of “permercurated ferrocene” with KBr_3. The assignment of signals is based on independent calculations of the isotope patterns.

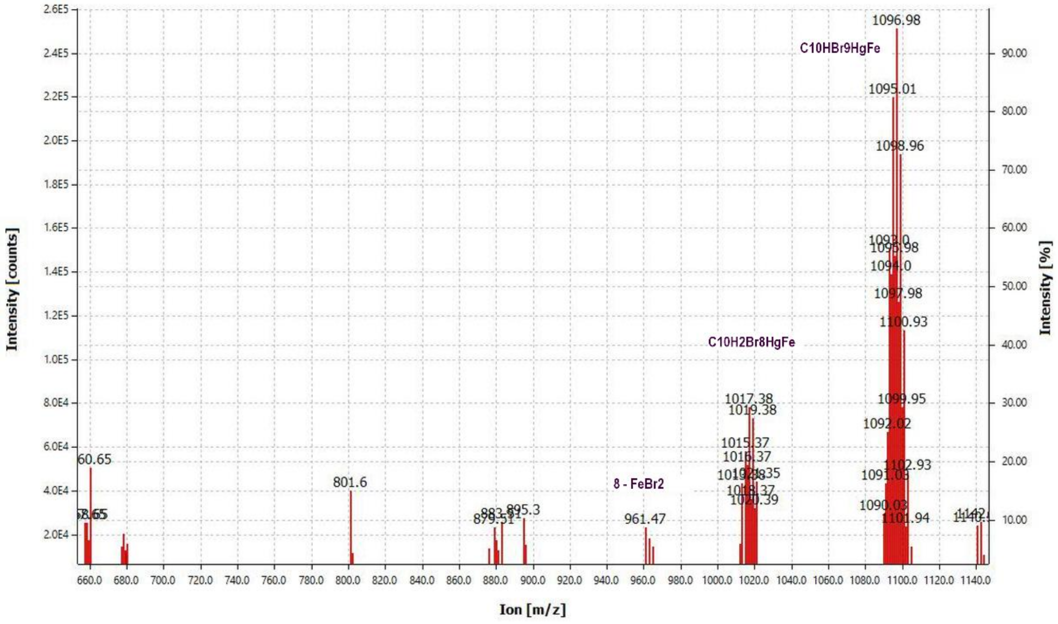

Figure S4 Partial EI mass spectrum (*m/z* > 650) of the second fraction from the reaction of “permercurated ferrocene” with KBr_3. The assignment of signals is based on independent calculations of the isotope patterns

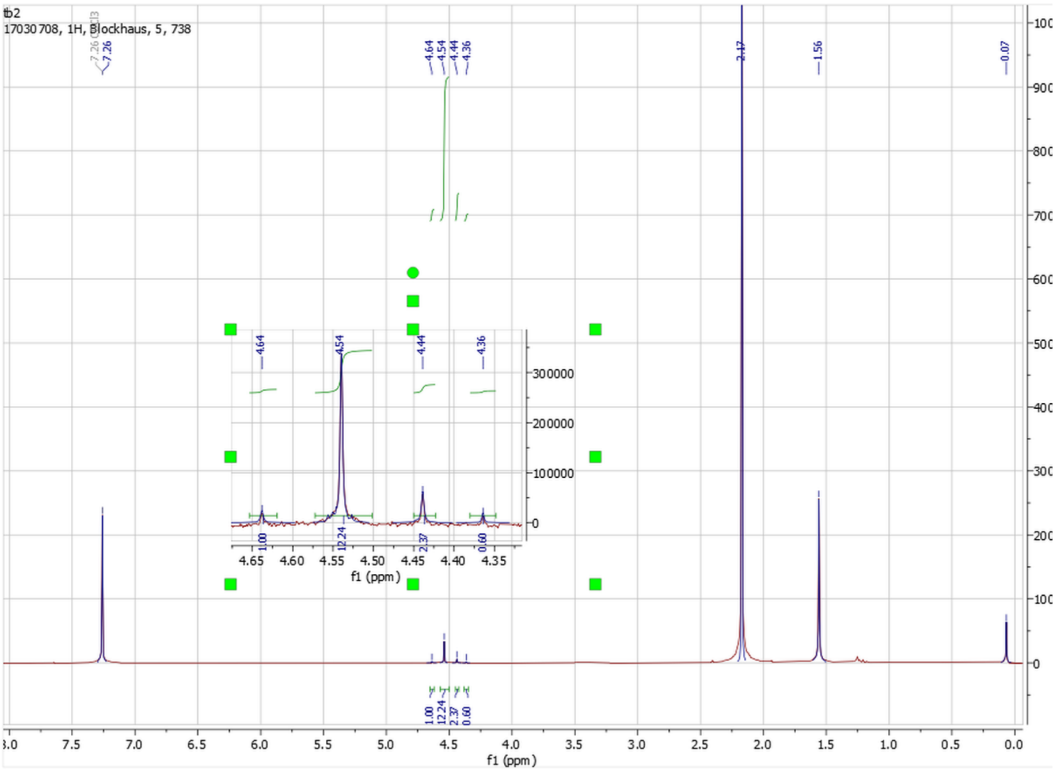

Figure S5 ^{1}H-NMR spectrum ($CDCl_3$) of the first fraction from the reaction of “permercurated ferrocene” with KBr_3.

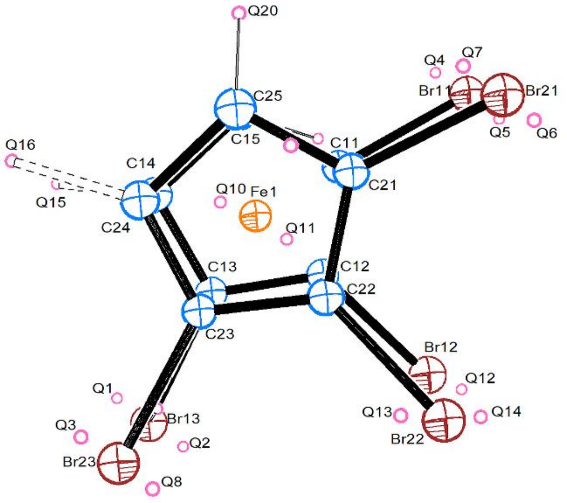

Figure S6 Top view of the result of the difference Fourier synthesis after the first refinement of the structural model obtained from SHELXT (ORTEP3 plot with displacement ellipsoids at the 30% probability level)

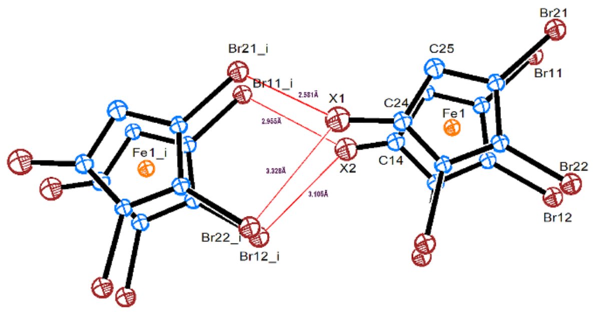

Figure S7 Top view (ortep3) of two translation related molecules after the first refinement cycles, showing the interaction of the residual electron density maxima X1 and X2 (corresponding to Q15 and Q16 of Figure S6) with bromine atoms Br11_i, Br12_1, Br21_i and Br22_i of a neighbouring molecule.

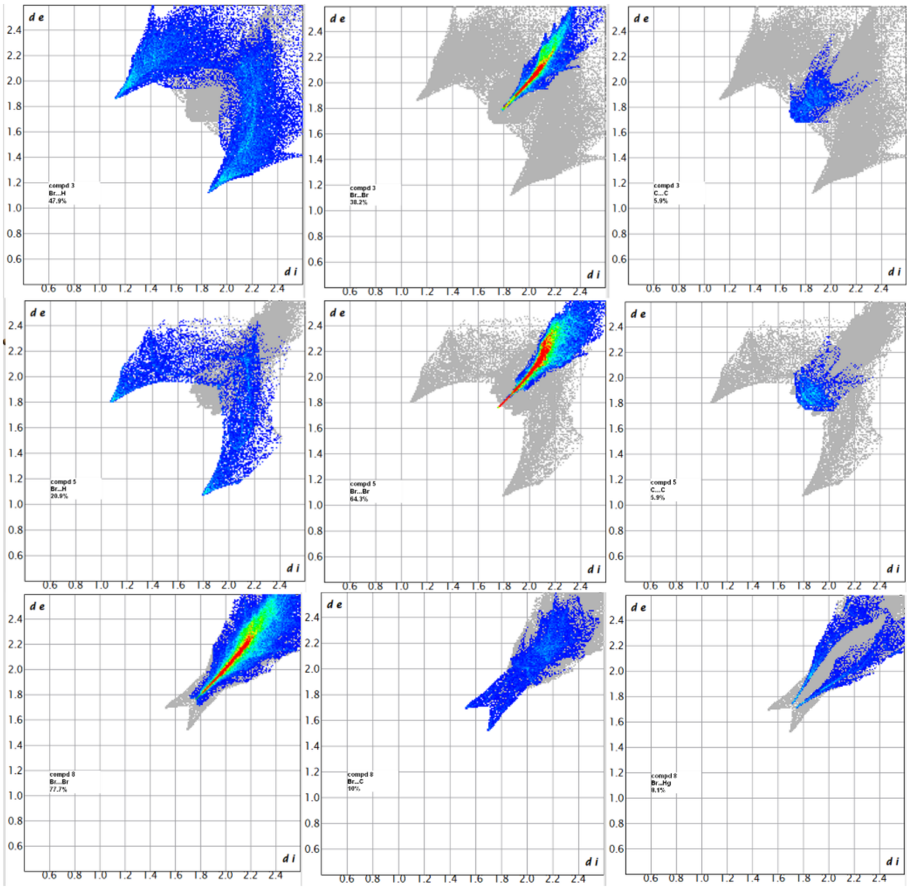

Figure S8 Individual fingerprint plots of compound **3** (top), **5** (middle) and **8** (bottom). Left: Br…H (**3** and **5**) and Br…Br (**8**); 2nd column: Br…Br (**3** and **5**) and Br…C (8); right column: C…C (**3** and **5**) and Br…Hg (**8**)

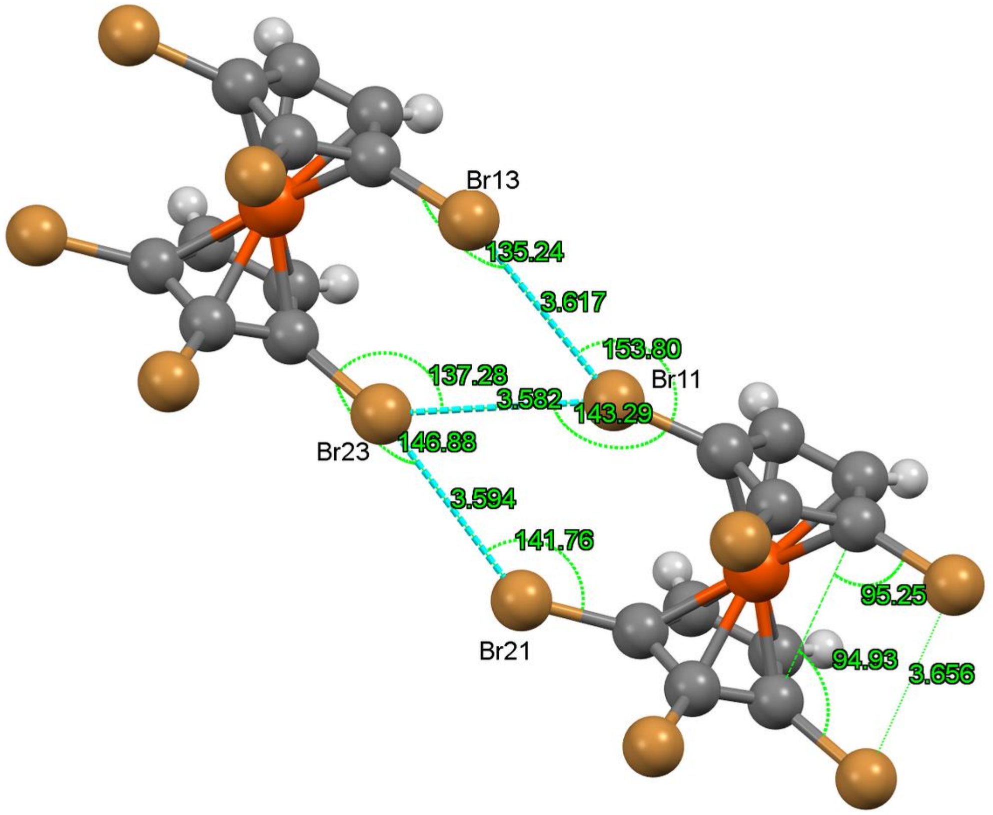

Figure S9 MERCURY representation of the geometrical parameters of the Br…Br contacts in **3**. Colour coding: carbon dark grey, hydrogen light grey, bromine brown, iron orange; blue lines are expanded contacts.

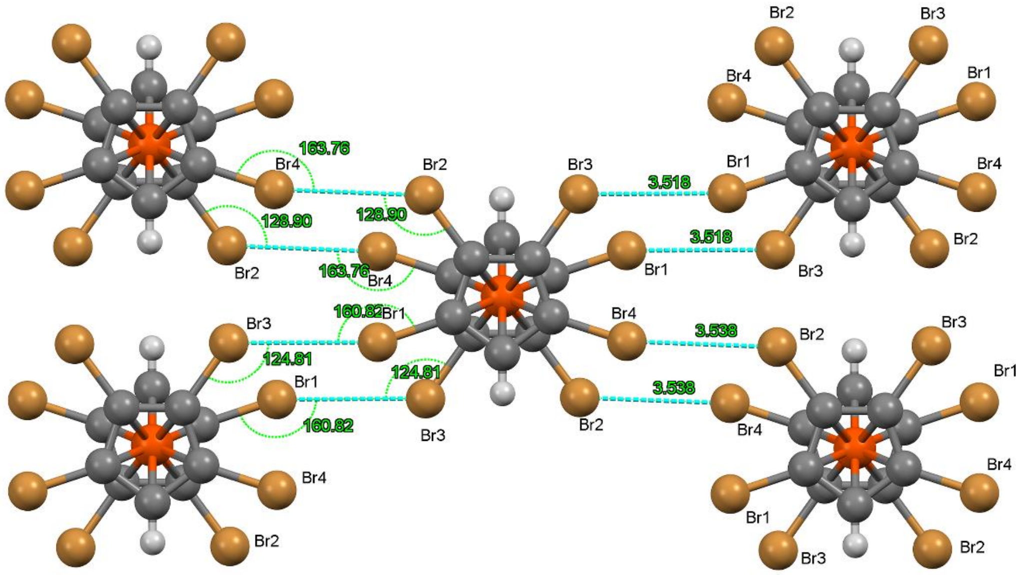

Figure S10 MERCURY representation of the geometrical parameters of the Br…Br contacts in **5.** For colour coding, see Figure S9)

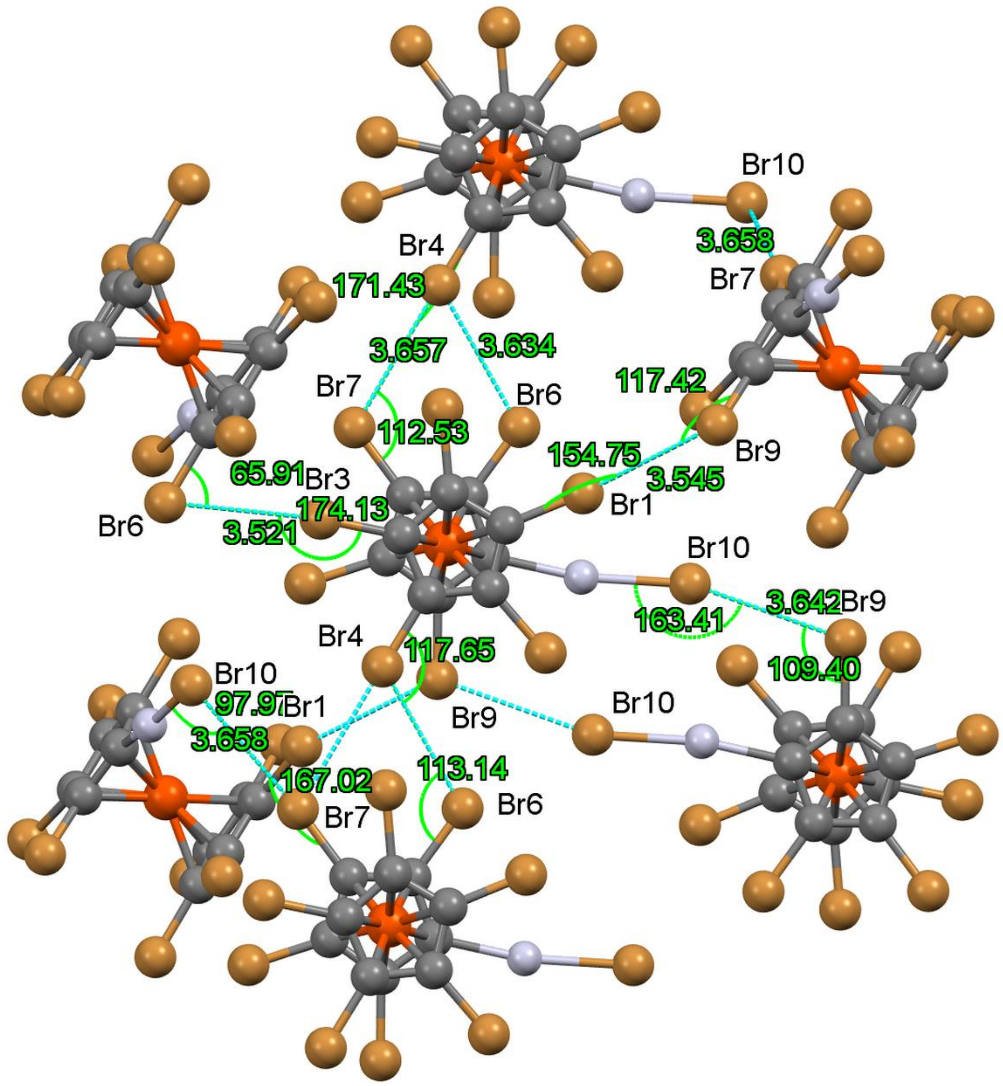

Figure S11 MERCURY representation of the geometrical parameters of the Br…Br contacts in **8.** For colour coding, see Figure S9. Additionally: Hg is light blue
